# Optimal acupuncture protocol improving symptoms of typical dry eye syndrome: meta-analysis and systematic review

**DOI:** 10.1016/j.heliyon.2023.e18226

**Published:** 2023-07-13

**Authors:** Joon-Gon Park, Bong Hyo Lee, Ji-Ho Na, Ji-Hyeo Jung, Chang-Hyun Song

**Affiliations:** a College of Korean Medicine, Daegu Haany University, Gyeongsan, 38610, Republic of Korea; b Department of Acupuncture, Moxibustion and Acupoint, College of Korean Medicine, Daegu Haany University, Daegu, 42158, Republic of Korea; c Department of Anatomy and Histology, College of Korean Medicine, Daegu Haany University, Gyeongsan, 38610, Republic of Korea

**Keywords:** Artificial tear, Acupuncture protocol, Keratoconjunctivitis sicca, Meridian, Schirmer, Tear-film breakup time, Xerophthalmia

## Abstract

Previous meta-analyses have shown a superiority of acupuncture over artificial tear for treating typical dry eye syndrome (DES). However, given that the acupuncture protocols were quite diverse in the randomized controlled trials (RCTs) included in the meta-analyses, it is necessary to establish the acupuncture guidelines. Thus, the optimal acupuncture protocol involved in improvements of tear-film breakup time (BUT) or Schirmer tear test (STT) was examined by meta-analyses for RCTs in patients with typical DES. Eight databases until Jun 2018 were searched for 21 RCTs (n = 1542 eyes) comparing effectiveness of acupuncture versus artificial tear control. Indirect comparison of Bucher analysis was used to find specific acupoints (SAPs) improving BUT or STT by comparing the outcomes between subgroups of the RCTs including and excluding certain SAPs. Meta-analysis was examined for the outcomes in subgroups of the RCTs based on the number of SAPs, and network meta-analysis was for multiple pairwise comparisons across the protocols using the SAPs to yield relative effects. The Bucher analyses identified nine SAPs with positive effects on BUT or STT, and the positive relations of two SAPs involved in improvements of both BUT and STT suggested potential combinations of three (‘KI3–LI4–SP6’ or ‘KI3–GB14–ST2’) or four SAPs (‘KI3–BL1–EX-HN7–SP6’). Subgroup meta-analyses showed the SAP-depending improvements of BUT or STT in the subgroups including more than three SAPs, compared with the artificial tear control. Meta-regression and network meta-analyses revealed significant correlations between the number of SAPs and the improvements of BUT and STT, and demonstrated that acupuncture using four SAPs for 21–30 days, particularly at two–three times per week, can be optimal for improving the symptoms of typical DES. These results provide useful information for guiding acupuncture in clinical trials for DES.

## 1 Introduction

Dry eye syndrome (DES) is a chronic ocular surface disease with a prevalence of 5%–50% worldwide, particularly predominant in aged population (i.e., 75% among adults ≥40 years) and females [1]. The symptoms are eye dryness, irritation, fatigue, and redness with a sensation of grittiness and burning, which eventually impairs a quality of life in the patients [2]. Age-related DES is an aqueous-deficient dry eye characterized by hyperosmolarity involving reduced lacrimal secretion with normal tear evaporation, and it is the most common form of non-Sjögren's syndrome [3]. The global occurrence is currently rising, along with increasing risk factors including uses of contact lenses and refractive surgery, and exposure to video display screens and dry environments, as well as aging [4]. The first-line treatment is a supplementation of artificial tear as a lubricant for the tear deficiency [2]. While the artificial tear relieves the symptoms of DES by reducing tear-film hyperosmolarity and diluting the inflammatory mediators, it requires frequent applications due to the temporary effectiveness. Eye drops of artificial tear containing anti-inflammatory agents (e.g., cyclosporine and corticosteroids) or autologous serum have shown favorable effects even on severe symptoms of DES; however, the long-term use arouses concerns of adverse effects (e.g., cataract) or cost-effectiveness [5]. The patients dissatisfied with the treatments try to seek other remedies, leading to growing interests in complementary medicines.

Acupuncture has been a major intervention to treat various diseases for several thousands of years in Traditional Chinese Medicine (TCM) [6]. It is known to be effective in some eye diseases, such as acute conjunctivitis, central retinitis, myopia, and cataracts [7]. Recent meta-analyses provide evidences that acupuncture alleviates symptoms of DES greater than artificial tears [[8], [9], [10], [11], [12], [13], [14]]. However, the number of randomized controlled trials (RCTs) included in the meta-analyses was insufficient to conclude the effectiveness. Our previous meta-analysis for 21 RCTs in patients with typical DES (without specific etiologies) has shown the superiority of acupuncture over artificial tear control in improving the symptoms [15]. On the other hand, the acupuncture protocols applied in the RCTs were quite diverse, thereby it is necessary to establish and standardize the acupuncture protocol for clinical reproducibility in DES.

A selection of the potential acupoint combinations is the most important element in the acupuncture protocol; however, DES-related meridians and the specific acupoints are unclear. In TCM, the human body is divided into 12 main *Yang* and *Yin* meridians referred to as internal organs, and human health is maintained by a flow of blood and energy (*Qi*) throughout the meridians [16]. Acupuncture targets certain acupoints on the meridians to correct an imbalance between *Yang* and *Yin* energies through local and systemic regulations [17]. DES is considered a *Yin* deficiency in the liver and kidney, which is a weak state to counterbalance *Yang* energy. Two *Yin* meridians of Liver (LR) and Kidney (KI) and the coupled *Yang* meridians of Bladder (BL) and Gallbladder (GB) are known to have a great influence on energy flows in the eye [18,19]. However, given that modern lifestyle and health status have changed completely from the previous eras, the traditional acupuncture protocol should be examined with scientific evidences for treating DES as a modern disease. There have been three meta-analyses reporting the effectiveness of acupoints in DES; however, some results were inconsistent. Kim et al. [12] reported that acupuncture on periocular ‘Stomach (ST)2’ is effective in DES, but acupuncture on the other periocular ‘BL2 and ST1’ are less effective; Lin et al. [13] suggested that acupuncture on periocular ‘ST1 and Triple energizer (TE)23’ and distant ‘Large intestine (LI)4’ can be optimal for treating DES; Wei et al. [14] reported that acupuncture using both periocular and distant body acupoints is more effective than that using periocular acupoints alone in DES. However, the meta-analyses included non-RCT [20], RCTs in patients with Sjögren's syndrome or refractive surgery [21,22], or RCTs with other interventions or non-traditional acupuncture (e.g., electro-/laser-acupuncture and acupuncture on non-acupoints) [[23], [24], [25], [26]]. Thus far, there have been no studies reporting the optimal acupuncture protocol using potential acupoints to treat typical DES.

Symptoms of tear-film stability and tear capacity in DES are usually diagnosed using the tear-film breakup time (BUT) and Schirmer tear test (STT), respectively, as the primary objective measurements, while the signs are assessed by subjective measurements including ocular surface disease index (OSDI) and visual analogue scale (VAS) [1]. However, results of the subjective self-assessments are not always consistent with those of the objective measurements [27]. Thus, these comprehensive meta-analyses were examined to evaluate the optimal acupuncture protocol focused on improvements of BUT and STT in the same 21 RCTs as reported previously [15]. Since there are a few studies comparing differential effects of a single acupoint on the symptoms of DES, indirect comparisons of Bucher analysis and Bayesian network meta-analysis were conducted to identify the related specific acupoints [12] and to examine multiple pairwise comparisons across the protocols including the specific acupoints, respectively [[28], [29], [30]]. Meta-regression analyses were examined for correlations of the specific acupoint with improvements of the primary outcomes, and publication bias and sensitivity analyses were for more rigorous quality control of these meta-analyses.

## 2 Methods

### 2.1 Literature search and selection

Acupuncture studies for treating DES were analyzed in the same literatures included in Na et al. [15]. The studies were searched according to the Preferred Reporting Items for Systematic Reviews and Meta-Analyses (PRISMA) guidelines [31]. Eight databases (PubMed, Medline, Web of Science, Cochrane, CNKI, Wangfang, OASIS, and RISS) were used to search the literature reported until June 2018. There were no language restrictions. The following key words were used: (‘acupuncture’) AND [(‘dry eye’) OR (‘xerophthalmia’) OR (‘keratoconjunctivitis sicca’)] AND [(‘treatment’) OR (‘alternative medicine’) OR (‘complementary medicine’)]. The studies were selected according to the following eligibility criteria: (1) RCTs in patients with typical DES; (2) RCTs comparing outcomes between acupuncture experimental group treated with acupuncture alone or acupuncture combined with artificial tear and the control treated with artificial tear; (3) acupuncture trials through a needle-insertion on active acupoints; (4) RCTs comparing at least two primary outcomes of BUT and STT. However, RCTs in DES patients with systemic disease or other causes, with other interventions (e.g., herbs and moxibustion), or with unclear data and information, were excluded. Furthermore, the relevant English-literatures searched through databases of Pubmed, Medline, Web of Science, and Cochrane until March 2022 were reviewed for the acupuncture protocol.

### 2.2 Data extraction

The risk of bias (ROB) for BUT and STT in 21 RCTs were as follows [15]: 36.8% low and 63.2% unclear ROB of random sequence generation; 10.5% low and 89.5% unclear ROB of allocation concealment; 100% low ROB of blinding; 80% low and 20% high ROB of incomplete data; 100% low ROB of selective outcome reporting; 80% low and 20% unclear ROB of other sources of bias according to descriptions of baseline data in the patients. In the acupuncture experimental and control groups, the outcomes of BUT and STT were expressed as the means and standard deviations in the post-to the pre-treatment with the sample sizes of eyes measured.

### 2.3 Statistical analysis

R version 3.4.1 (The R Foundation, Vienna, Austria) was used for statistical analyses, as described previously [15]. Heterogeneity across the studies was determined graphically and statistically using forest plots and Higgins *I*^2^ tests, respectively [32,33]. A random-effects model was used for the meta-analyses because of the substantial heterogeneity in all data (*P* < 0.10, *I*^2^ > 50%). The results are expressed as the mean differences (MDs) with 95% confidence intervals (CIs), and the MDs were weighted by the inverse variances. The data were considered significantly different when 95% CIs did not cross the cutoff point of zero. Sensitivity analysis was performed by leaving each RCT out sequentially and comparing the model characteristics [34]. Publication bias was assessed by a visual inspection of the funnel plots, and the asymmetry was adjusted using the trim and fill method [35]. Meta-regression analysis was examined for correlations between acupuncture protocols and outcomes of BUT or STT, and the results were expressed as the coefficients with the proportion of variance in the outcomes (*R*^2^). *P* values < 0.05 were considered significant. Bucher's method was performed by comparing MDs of the outcomes in the experimental group versus the control between the subgroups of RCTs including and excluding certain acupoints [36]. Furthermore, Bayesian network meta-analysis was performed to synthesize the direct and indirect evidence for the optimal acupuncture in DES, as described previously [28,30,37]. The Markov chain Monte Carlo (MCMC) method using the *rjags* and *gemtc* packages was used, and three MCMC chains were fitted, each with 50,000 burn-ins and 100,000 iterations. The pooled relative effect sizes were expressed as the posterior medians and 95% credible intervals (CrIs), and the positive values were regarded significantly different. Network diagram in the network meta-analysis was observed for the competing interventions, and results of the indirect model were compared with those of the direct model by determining whether the transitivity assumption was valid [38,39]. In addition, the distribution of probabilities ranking plots was examined for the optimal number of specific acupoints in acupuncture for treating DES.

## 3 Results

### 3.1 Acupuncture protocols in 21 RCTs

**Fig. 1:**
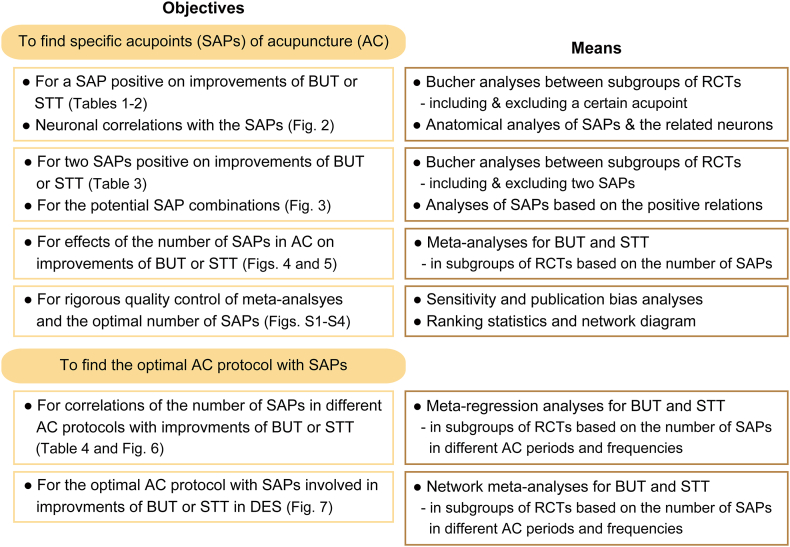
**A network diagram for fundamental objectives and means.** For the optimal acupuncture protocol involved in improvements of tear-film breakup time (BUT) or Schirmer tear test (STT) in dry eye syndrome (DES), each of the objectives were examined by the corresponding analyses in the subgroups of randomized controlled trials (RCTs).

The effects of acupuncture alone (#1–#14) or acupuncture combined with artificial tear (#15–#19) in the acupuncture experimental groups were compared with those of the artificial tear controls among 19 studies in patients with typical DES (Table 1). Because two studies (#1 and #12) were conducted with two independent trials, they were divided into ‘a’ and ‘b’ in each. Thus, 21 RCTs (1214 patients of 40% males and 60% females, aged 33–61 years) were included in these meta-analyses. The control group was treated with three–six drops (0.15–0.30 ml) of artificial tear once a day or the eye-drops three–six times a day. The experimental groups were treated with acupuncture at two–three (11 RCTs) or 3.7–7.0 times per week (10 RCTs) for 21–90 days (15 and 6 RCTs with acupunctures for up to 30 days and 51–90 days, respectively). For analyzing the acupuncture protocols, the RCTs were divided into high- and low-frequency subgroups based on the acupuncture session of 3.5-times per week, or short- and long-term subgroups based on the treatment period of 30 days. The acupuncture was applied on 31 acupoints of four extra-points in the head and neck (EX-HN) and 27 acupoints on 10 meridians. The acupoints are on 18 local (11 periocular and 7 head), and 13 distant (limbs) sites based on distances from the eye lesions. The distant points were LI4/11 and Lung (LU)9/10 on the upper-limb, and GB34/37, KI3, LR3, Spleen (SP)3/6/10 and ST36/40 on the lower-limb. Sixteen acupoints of BL1/2, EX-HN5/7, GB1/14/20, Governor (GV)20, KI3, LI4, LR3, SP6, ST1/2/36, and TE23 were applied in more than three RCTs, and their effects on improvements of BUT or STT were examined by comprehensive statistical analyses (Fig. 1).

**Table 1:** Randomized controlled trials (RCTs) of acupuncture for treating typical dry eye syndrome (DES).

Studies (#)	Treatments	BL			EX-			GB				GV		KI	LI		LR	LU	SP		ST				TE	
	Control & Experimental	1	2	3	3/4	5	7	1	14	20	37	20	23/26	3	4	11/20	3	9/10	3/9/10	6	1	2	36	40	17	23
1. He et al. [40]^†^	a (n = 20) 5T & (n = 20) 2.3S, 90D													3	4	11/20			-/9/10	6		2	36	40		
	b (n = 20) 5T & (n = 20) 2.3S, 90D		2						14												1					23
2. Zhang et al. [41]	(n = 30) 3–6T & (n = 30) 5.4S, 90D													3			3				1					
3. Zhang [42]	(n = 26) 6T & (n = 28) 6S, 28D	1	2			5		1																		23
4. Kim et al. [43]	(n = 75) > 1T & (n = 75) 3S, 28D		2			5			14	20			23/-		4	11/-					1					23
5. Shi et al. [44]	(n = 34) 3–4T & (n = 31) 3S, 21D	1				5						20			4						1		36			23
6. Li et al. [45]^†^	(n = 12) 3 drops & (n = 12) 6S, 28D	1	2			5		1																		23
7. Liu [46]	(n = 50) 3T & (n = 50) 7S, 28D		2		-/4	5				20		20			4					6	1					23
8. Wang [47]	(n = 28) 4T & (n = 28) 6.6S, 51D	1	2			5	7			20		20			4					6		2	36			
9. Chao [48]^†^	(n = 16) 4T & (n = 19) 7S, 28D		2			5				20	37			3			3			6		2	36			
10. Leng et al. [49]	(n = 29) 3–6T & (n = 29) 3S, 21D	1	2			5		1																		23
11. Mei [50]^†^	(n = 34) 4 drops & (n = 30) 3S, 30D	1	2		-/4	5								3	4			-/10			1					23
12. Ni et al. [51]	a (n = 31) 5T & (n = 30) 3S, 21D	1					7						-/26	3						6						
	b (n = 31) 5T & (n = 32) 3S, 21D	1					7							3						6						
13. Xiang et al. [26]	(n = 44) 4 drops & (n = 44) 3.7S, 21D	1	2																			2				23
14. Feng et al. [52]	(n = 33) 3–6 drops & (n = 33) 3S, 30D	1			3/-	5				20		20			4					6						
15. Tseng et al. [53]^†^	(n = 17) NR & (n = 9) 2S, 56D					5			14											6		2				23
16. Liu [54]	(n = 45) 4–5 drops & (n = 45) 7S, 28D	1	2			5				20		20										2				23
17. Li & Lu [55]^†^	(n = 16) 3–4T & (n = 17) 7S, 30D		2						14		37			3	4		3		-/-/10			2	36		17	23
18. Hu [56]	(n = 30) 4 drops & (n = 34) 7 S, 28D		2			5			14			20	-/26				3	9/-	3/−/−			2				
19. Liu et al. [57]^†^	(n = 14) NR & (n = 14) 3 S, 56D		2	3		5				20		20			4						1	2				23
Numbers of acupoints applied		11	14	1	1/2	14	3	3	5	7	2	7	1/2	7	9	2/1	4	1/1	1/1/2	8	7	9	5	1	1	13

Effectiveness of acupuncture experimental groups versus the artificial tear controls were compared in one or both eyes^†^ (n) of patients with typical DES. Treatments are shown as the number of eye-drops once a day (drops) or the eye-drops a day (times, T) in the control, and as the number of acupuncture sessions (S) a week for the treatment days (D) in the experimental group. *Abbreviations.* BL: Bladder, EX-: Extra-point in head & neck, GB: Gallbladder, GV: Governor vessel, KI: Kidney, LI: Large intestine, LR: Liver, LU: Lung, NR: not-reported, SP: Spleen, ST: Stomach, and TE: Triple energizer.

### 3.2 Specific acupoints involved in improvements of BUT or STT

Effects of a single acupoint on the outcomes of BUT or STT were compared between the subgroups of RCTs including and excluding one of 16 acupoints used in more than three RCTs by the Bucher analysis (Table 2). The subgroups including one of five acupoints, ‘BL1, EX-HN7, KI3, LI4, and SP6’, showed higher MDs of both BUT and STT in the experimental group versus the control, compared with those excluding the same one. The subgroups including one of another four acupoints, ‘GB14, GV20, ST2, and ST36’, showed higher MDs of STTs only in the experimental group versus the control, compared with those excluding the same one. However, the MDs of both BUT and STT were lower in the subgroups including one of the other seven acupoints than those excluding the same one. Nine acupoints with the positive effects on both BUT and STT (BUT/STT^+^) or STT only (STT^+^) were regarded as specific acupoints (SAPs) involved in improvements of BUT or STT.

**Table 2:** Subgroup analyses on a single specific acupoint (SAP) involved in improvements of BUT or STT.

Acupoints (regions)	In/Ex	Tear-film breakup time (BUT)			Schirmer tear test (STT)		
		MD [95% CI] (n, Con/Exp)		*I*^2^	MD [95% CI] (n, Con/Exp)		*I*^2^
*SAPs positive on both BUT and STT (BUT/STT*^*+*^*-SAPs)*							
BL1	In	↑	1.64 [0.79, 2.48] (393/384)	89.7	↑	2.50 [1.48, 3.52] (393/384)	89.7
(PO)	Ex		1.30 [0.47, 2.14] (371/392)	89.5		2.41 [1.07, 3.74] (371/394)	94.2
EX-7	In	↑	2.84 [0.07; 5.62] (90/90)	92.6	↑	3.93 [2.46; 5.40] (90/90)	58.5
(PO)	Ex		1.24 [0.73; 1.75] (674/686)	84.9		2.25 [1.29; 3.20] (674/686)	94.3
LI4	In	↑	1.64 [0.69, 2.60] (386/375)	88.5	↑	2.70 [1.61, 3.79] (386/375)	85.5
(UL)	Ex		1.35 [0.63, 2.07] (378/401)	89.2		2.30 [1.01; 3.58] (378/403)	95.7
KI3	In	↑	2.47 [1.18, 3.76] (262/260)	91.5	↑	3.05 [1.60, 4.51] (262/260)	88.9
(LL)	Ex		1.02 [0.43, 1.61] (502/516)	85.7		2.17 [1.05, 3.29] (502/518)	94.9
SP6	In	↑	2.09 [0.69, 3.49] (261/278)	70.1	↑↑	3.77 [2.83, 4.71] (261/280)	85.0
(LL)	Ex		1.05 [0.61, 1.48] (503/498)	94.6		1.64 [0.81, 2.47] (503/498)	86.4
***SAPs positive on STT only (STT***^***+***^***-SAPs)***							
GB14	In	↓	1.13 [0.29, 1.96] (191/206)	78.7	↑	3.10 [1.08, 5.12] (191/208)	95.3
(PO)	Ex		1.56 [0.83, 2.28] (573/570)	90.4		2.27 [1.41, 3.13] (573/570)	89.8
GV20	In	↓	1.26 [0.10, 2.42] (248/249)	90.4	↑	2.69 [1.69, 3.69] (248/249)	80.0
(Head)	Ex		1.57 [0.88, 2.26] (516/527)	89.1		2.38 [1.17; 3.59] (516/529)	95.4
ST2	In	↓	0.73 [0.15, 1.31] (293/314)	71.3	↑	2.66 [1.13, 4.18] (293/316)	95.8
(PO)	Ex		1.97 [1.16, 2.78] (471/462)	90.6		2.32 [1.30, 3.35] (471/462)	89.6
ST36	In	↓	1.12 [0.19, 2.05] (164/167)	71.3	↑	2.55 [1.32, 3.77] (164/167)	77.7
(LL)	Ex		1.58 [0.88, 2.28] (600/609)	91.2		2.41 [1.34, 3.48] (600/611)	94.8
***Acupoints negative on BUT and STT***							
BL2	In	↓	1.26 [0.58, 1.94] (393/384)	86.7	↓	2.09 [1.27, 2.90] (549/553)	87.2
(PO)	Ex		1.92 [0.75, 3.08] (371/392)	93.2		3.16 [1.57, 4.75] (215/225)	94.1
EX-5	In	↓	1.21 [0.60, 1.83] (90/90)	85.9	↓	2.42 [1.41, 3.43] (518/532)	92.8
(PO)	Ex		2.07 [0.69, 3.45] (674/686)	93.5		2.57 [0.91, 4.24] (246/246)	93.6
GB1	In	↓	1.30 [0.76, 1.84] (262/260)	0.0	↓	1.11 [0.48, 1.74] (79/81)	10.8
(PO)	Ex		1.51 [0.84, 2.18] (502/516)	90.8		2.60 [1.64, 3.56] (685/697)	93.9
ST1	In	↓	1.35 [0.30, 2.40] (191/206)	91.5	↓	1.48 [0.13, 2.83] (325/314)	90.3
(PO)	Ex		1.54 [0.82, 2.27] (573/590)	88.6		2.98 [1.84, 4.11] (439/464)	94.4
TE23	In	↓	1.25 [0.58, 1.91] (164/167)	88.5	↓	2.21 [1.04; 3.38] (509/513)	95.3
(PO)	Ex		1.86 [0.71, 3.00] (600/609)	90.2		2.89 [1.59, 4.19] (255/265)	87.2
GB20	In	↓	1.10 [-0.04, 2.23] (386/375)	90.7	↓	2.19 [1.02, 3.37] (291/297)	85.5
(Head)	Ex		1.65 [0.96, 2.35] (378/401)	89.2		2.62 [1.44, 3.80] (473/481)	95.1
LR3	In	↓	1.38 [0.37, 2.39] (261/278)	76.0	↓	2.17 [-0.18, 4.53] (122/132)	91.0
(LL)	Ex		1.48 [0.80, 2.17] (503/498)	90.7		2.53 [1.56, 3.50] (642/646)	94.1

Outcomes of the experimental groups (Exp) versus the controls (Con) were compared between subgroups including (In) and excluding (Ex) each acupoint. Arrows mean the higher or lower mean differences (MDs) between the subgroups. Double arrows are for the MDs without overlapping 95% confidence intervals (CIs). *Abbreviations*. LL: lower-limb, PO: periocular region, and UL: upper-limb.

### 3.3 Anatomical locations of SAPs and the neuronal correlations

**Fig. 2:**
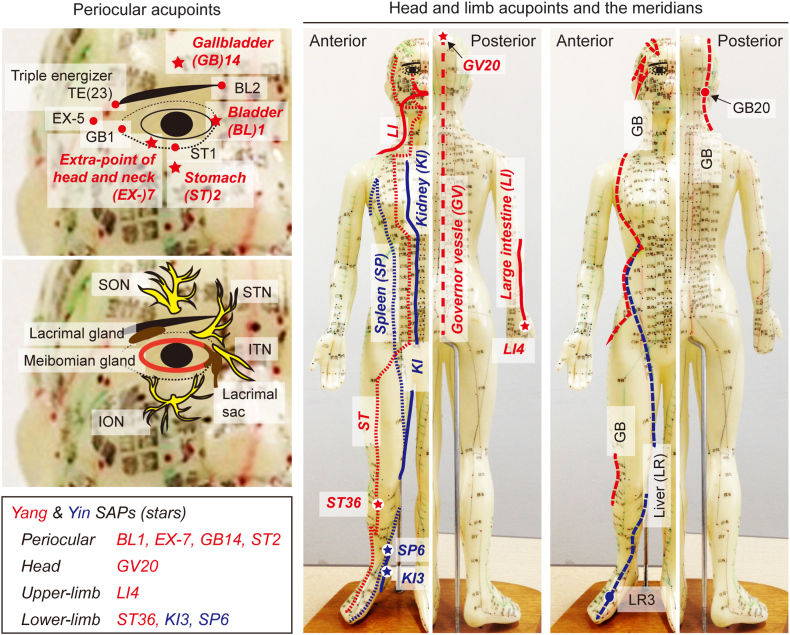
**Anatomical locations of acupoints and the trigeminal nerve innervations.** Sixteen acupoints on *Yang* (red) and *Ying* (blue) meridians, included in these meta-analyses, were indicated according to the anatomical locations in the periocular region, head and limbs. Nine specific acupoints (SAPs) involved in improvements of tear-film breakup time (BUT) or Schirmer tear test (STT), are listed. The trigeminal nerve branches correlated with the periocular SAPs, the supra-/infra-orbital nerve (SON/ION) and the supra-/infra-trochlear nerve (STN/ITN), are depicted with the lacrimal system. (For interpretation of the references to colour in this figure legend, the reader is referred to the Web version of this article.)

Nine SAPs were located as follows (Fig. 2): ‘BL1’ is in the depression superior to the inner canthus; ‘EX-HN7’ is at the junction of the lateral one-fourth and the medial three-fourths of the infraorbital margin; ‘GB14’ is on the forehead directly above the pupil at the width of two fingers above the midpoint of the eyebrow; ‘ST2’ is directly below the pupil, in the depression over the infraorbital foramen; ‘GV20’ is at the top of the head, over the sagittal suture; ‘LI4’ is on the dorsal hand between the 1st and 2nd metacarpal bones; ‘KI3’ is in the depression between the medial malleolus and the Achilles tendon; ‘SP6’ is on the anterior four finger widths directly above the tip of the medial malleolus on the posterior border of the tibia; ‘ST36’ is in the tibialis anterior muscle at the four finger widths below the patella and the one finger width lateral from the anterior tibial crest [58]. The local SAPs are known to correlate with the trigeminal ophthalmic (V1) or maxillary (V2) nerve branches as follows [59,60]: ‘BL1’ targets the palpebral branch of the infratrochlear nerve (n.) from the nasociliary n. In V1; ‘GB14’ targets the common calvarial branch of the supraorbital n. From the frontal n. In V1; ‘GV20’ is located at the sensory region of the frontal n. In V1; ‘ST2’ and ‘EX-HN7’ target the infraorbital n. And the lateral branch of the inferior palpebral n. From the infraorbital n., respectively, in V2. However, the other five periocular acupoints with the negative effects on both BUT and STT showed correlations with the different trigeminal nerve branches: ‘BL2’ and ‘TE23’ target the supratrochlear n. From the frontal n. And the lacrimal n., respectively, in V1; ‘EX-HN5 and GB1’ target the zygomatic n. In V2; ‘ST1’ targets the medial branch of the inferior palpebral n. From the infraorbital n. In V2. In the distant SAPs, ‘LI4’ is covered by the superficial branch of the radial n. [61], while ‘KI3 and SP6’ and ‘ST36’ are correlated with the posterior tibial n. And the deep peroneal n., respectively [62,63].

### 3.4 SAP combinations involved in improvements of BUT or STT

**Fig. 3:**
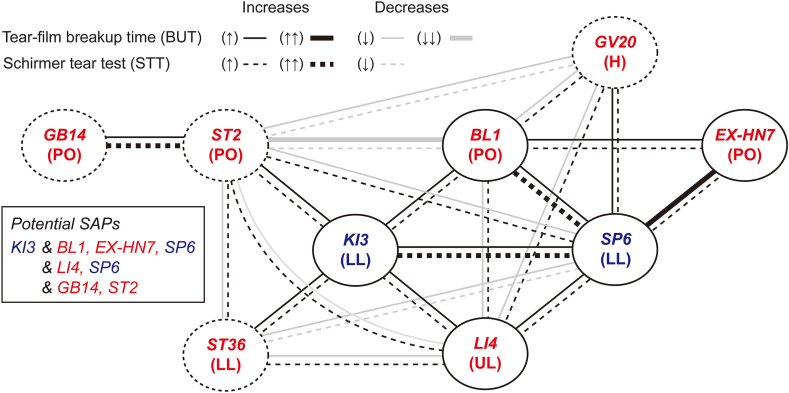
**Potential SAP combinations involved in improvements of BUT or STT.** Nine SAPs with positive effects on both BUT and STT (lined circles) or STT only (dotted-circles), are indicated. Lines and dotted-lines show the positive (black) or negative (grey) effects of two SAPs on improvements of BUT and STT, respectively. The SAPs connected only with black lines and dotted-lines are listed as the potential SAP combinations. H: head, LL: lower-limb, PO: periocular, and UL: upper-limb.

Effects of two SAPs on the outcomes of BUT and STT were further compared by Bucher analysis (Table 3). The indirect comparisons were available between the subgroups of RCTs including and excluding one of 21 combinations with two SAPs. Positive effects on the outcomes of both BUT and STT in the experimental group versus the control were found in the subgroups including one of the following 11 combinations compared with those excluding the same one: seven combinations with two BUT/STT^+^-SAPs, excepting ‘BL1 & LI4’, and four combinations with each of BUT/STT^+^- and STT^+^-SAPs (‘KI3 & ST2 or ST36’ and ‘SP6 & GV20’) or two STT^+^-SAPs (‘ST2 & GB14’). However, the negative effects on both BUT and STT were found in the subgroups including one of three combinations with each of BUT/STT^+^- and STT^+^-SAPs (‘BL1 & ST2 and SP6 & ST36’) or two STT^+^-SAPs (‘ST2 & GV20’), compared with those excluding the same one. The subgroups including one of the other seven combinations showed the positive effects on STT only. Among 11 combinations involved in improvements of both BUT and STT, two and four combinations had two local and two distant SAPs, respectively, and the other five had each of the local and distant SAPs. Among seven combinations involved in improvements of STT only, only two had two local or two distant SAPs, and five had each of the local and distant SAPs. Two and one combinations with the negative effects on both BUT and STT, had two local and two distant SAPs, respectively. Particularly, the strong positive effects with no overlapping 95% CIs were observed in the BUT between the subgroups including and excluding two BUT/STT^+^-SAPs of the periocular ‘EX-HN7’ & the distant ‘SP6’, and in the STT between the subgroups including and excluding two BUT/STT^+^-SAPs of the periocular ‘BL1’ & the distant ‘KI3’ or ‘SP6’ or two STT^+^-SAPs of the periocular ‘ST2’ & ‘GB14’. The potential combinations involved in the improvements of both BUT and STT were ‘KI3–LI4–SP6’ and ‘KI3–GB14–ST2’ as three SAPs, and ‘KI3–BL1–EX-HN7–SP6’ as four SAPs, based on the positive relations (Fig. 3).

**Table 3:** Subgroup analyses on two SAPs involved in improvements of BUT or STT.

Acupoints (regions)	In/Ex	Tear-film breakup time (BUT)			Schirmer tear test (STT)		
		MD [95% CI] (n, Con/Exp)		*I*^2^	MD [95% CI] (n, Con/Exp)		*I*^2^
Two BUT/STT^+^-SAPs							
BL1 (PO)	In	**↑**	2.84 [0.07, 5.62] (90/90)	92.6	**↑**	3.93 [2.46, 5.40] (90/90)	58.5
& EX-7 (PO)	Ex		1.24 [0.73, 1.75] (674/686)	84.9		2.25 [1.29, 3.20] (674/688)	94.3
BL1 (PO)	In	**↑**	2.97 [1.05, 4.90] (162/160)	93.2	**↑**	3.54 [2.08, 5.00] (162/160)	82.9
& KI3 (LL)	Ex		1.13 [0.60, 1.65] (602/616)	84.3		2.20 [1.17, 3.22] (602/618)	94.5
BL1 (PO)	In	**↑**	2.58 [0.52, 4.64] (123/123)	92.0	**↑↑**	4.16 [3.43, 4.90] (123/123)	38.4
& SP6 (LL)	Ex		1.21 [0.68, 1.74] (641/653)	85.4		2.13 [1.14, 3.12] (641/655)	94.5
EX-7 (PO)	In	**↑↑**	3.62 [2.89, 4.34] (90/90)	92.6	**↑**	4.10 [3.17, 5.02] (90/90)	58.5
& SP6 (LL)	Ex		1.02 [0.83, 1.21] (674/686)	84.9		2.25 [1.29, 3.20] (674/688)	94.3
LI4 (UL)	In	**↑**	1.87 [0.12, 3.62] (151/151)	90.5	**↑**	3.74 [3.17, 4.30] (151/151)	22.3
& SP6 (LL)	Ex		1.36 [0.79, 1.94] (613/625)	87.3		2.22 [1.18, 3.25] (613/627)	94.5
LI4 (UL)	In	**↑**	2.44 [1.78, 3.10] (138/130)	31.5	**↑**	3.68 [3.01, 4.35] (138/230)	0.0
& KI3 (LL)	Ex		1.32 [0.70, 1.94] (626/646)	89.9		2.21 [1.23, 3.20] (626/648)	94.3
KI3 (LL)	In	**↑**	3.51 [1.62, 5.40] (102/102)	88.0	**↑↑**	4.03 [3.30, 4.76] (102/102)	20.3
& SP6 (LL)	Ex		1.15 [0.63, 1.67] (662/674)	85.1		2.17 [1.20, 3.14] (662/676)	94.2
BL1 (PO)	In	**↓**	1.29 [0.19, 2.40] (163/152)	80.2	**↑**	2.83 [1.34, 4.32] (163/152)	83.4
& LI4 (UL)	Ex		1.53 [0.85, 2.21] (601/624)	90.6		2.38 [1.35, 3.41] (601/626)	94.5
***BUT/STT***^***+***^***-SAP & STT***^***+***^***-SAP***							
KI3 (LL)	In	**↑**	1.72 [0.24, 3.20] (102/108)	79.1	**↑**	3.21 [1.42, 4.99] (102/108)	82.7
& ST2 (PO)	Ex		1.41 [0.79, 2.03] (662/668)	89.7		2.33 [1.34, 3.31] (662/670)	94.3
KI3 (LL)	In	**↑**	1.72 [0.24; 3.20] (102/108)	79.1	**↑**	3.21 [1.42, 4.99] (102/108)	82.7
& ST36 (LL)	Ex		1.41 [0.79; 2.03] (662/668)	89.7		2.33 [1.34, 3.31] (662/670)	94.3
SP6 (LL)	In	**↑**	1.76 [-0.28, 3.80] (111/111)	91.1	**↑**	3.86 [3.18, 4.53] (111/111)	42.0
& GV20 (H)	Ex		1.38 [0.83, 1.93] (653/665)	86.7		2.29 [1.31, 3.27] (653/667)	94.2
BL1 (PO)	In	**↓**	0.83 [0.13, 1.52] (140/137)	55.6	**↑**	2.51 [1.24, 3.79] (140/137)	79.4
& GV20 (H)	Ex		1.62 [0.95, 2.30] (624/639)	90.3		2.45 [1.40, 3.50] (624/641)	94.6
LI4 (UL)	In	**↓**	1.07 [-0.55, 2.69] (126/126)	81.4	**↑**	2.81 [1.11, 4.51] (126/126)	74.1
& ST2 (PO)	Ex		1.53 [0.91, 2.14] (638/650)	89.9		2.39 [1.40, 3.39] (638/652)	94.6
LI4 (UL)	In	**↓**	1.18 [-0.29, 2.64] (17/1703)	91.4	**↑**	2.51 [1.00, 4.02] (173/170)	85.6
& GV20 (H)	Ex		1.51 [0.90, 2.12] (591/606)	87.8		2.46 [1.40, 3.52] (591/608)	94.7
LI4 (UL)	In	**↓**	1.31 [0.05; 2.56] (132/129)	76.2	**↑**	2.87 [1.27, 4.46] (132/129)	80.6
& ST36 (LL)	Ex		1.48 [0.84; 2.12] (632/647)	90.1		2.36 [1.35, 3.38] (632/649)	94.6
SP6 (LL)	In	**↓**	0.51 [-0.21, 1.23] (116/133)	55.6	**↑**	3.13 [1.27, 4.99] (116/135)	93.5
& ST2 (PO)	Ex		1.67 [1.02, 2.31] (648/643)	89.2		2.30 [1.44, 3.16] (648/643)	90.1
BL1 (PO)	In	**↓↓**	0.19 [-0.27, 0.66] (117/117)	2.4	**↓**	1.48 [-0.35, 3.30] (117/117)	90.7
& ST2 (PO)	Ex		1.67 [1.06, 2.29] (647/659)	88.8		2.63 [ 1.71, 3.55] (647/661)	92.6
SP6 (LL)	In	**↓**	0.69 [-0.40; 1.78] (100/106)	58.9	**↓**	2.43 [1.10, 3.76] (100/106)	70.0
& ST36 (LL)	Ex		1.58 [ 0.96; 2.20] (664/670)	89.8		2.47 [1.48, 3.47] (664/672)	94.4
***Two STT+-SAPs***							
ST2 (PO)	In	**↑**	1.64 [-0.15, 3.42] (76/91)	89.0	**↑↑**	4.63 [3.62, 5.64] (76/93)	55.4
& GB14 (PO)	Ex		1.43 [0.81, 2.04] (688/685)	88.7		2.12 [1.34, 2.90] (688/685)	89.1
ST2 (PO)	In	**↓**	1.25 [-0.11, 2.60] (130/136)	77.6	**↑**	2.95 [1.53, 4.36] (130/136)	74.9
& ST36 (LL)	Ex		1.49 [0.87, 2.12] (634/640)	90.0		2.34 [1.32, 3.36] (634/642)	94.6
ST2 (PO)	In	**↓**	0.53 [-0.39, 1.45] (131/135)	60.7	**↓**	2.35 [1.26, 3.43] (131/135)	57.3
& GV20 (H)	Ex		1.66 [1.02, 2.30] (633/641)	90.1		2.52 [1.49, 3.55] (633/643)	94.7

Outcomes of the experimental groups (Exp) versus the controls (Con) were compared between subgroups including (In) and excluding (Ex) each of two SAPs. Arrows mean the higher or lower MDs between the subgroups. Double arrows are for the MDs without overlapping 95% CIs.

### 3.5 SAP-depending improvements of BUT or STT

**Fig. 4:**
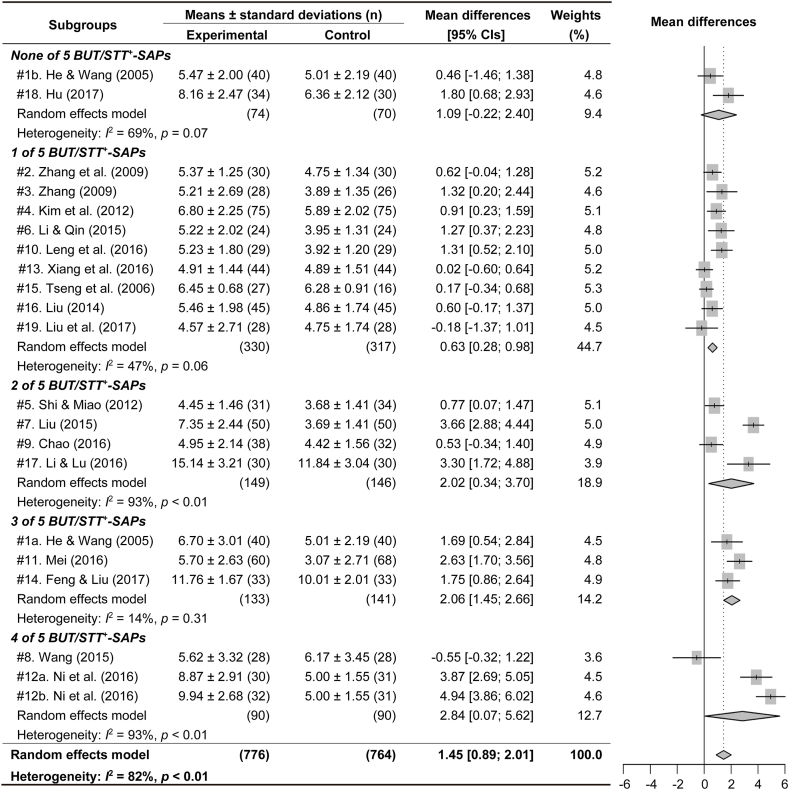
**Forest plots for meta-analysis of BUT in subgroup based on the number of SAPs.** Twenty-one RCTs were divided into subgroups including none to four of five SAPs with positive effects on both BUT and STT (BUT/STT^+^-SAPs). The subgroup meta-analysis was examined for the outcomes of BUT in the acupuncture experimental groups versus the controls after the treatments.

**Fig. 5:**
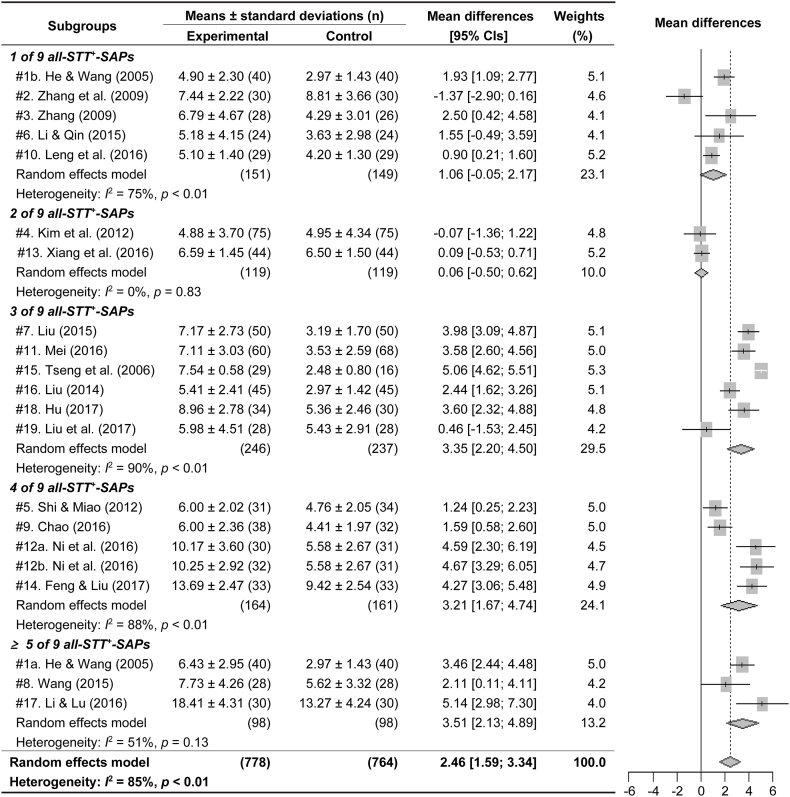
**Forest plots for meta-analysis of STT in subgroups based on the number of SAPs.** Twenty-one RCTs were divided into subgroups including one to four and more than five of all of nine SAPs with positive effects on the STT (all-STT^+^-SAPs). The subgroup meta-analysis was examined for the outcomes of STT in the experimental groups versus the controls after the treatments.

**Fig. 6:**
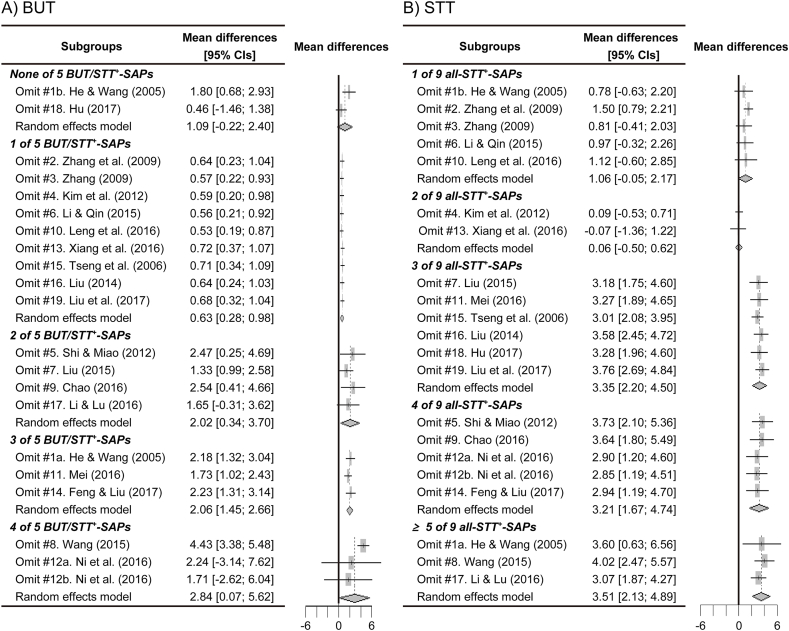
**Sensitivity analyses for BUT or STT in the subgroups based on the number of SAPs.** Sensitivity analyses were performed in the same subgroups in Fig. 4, Fig. 5 for comparing the outcomes of meta-analyses for BUT (A) and STT (B), respectively.

**Fig. 7:**
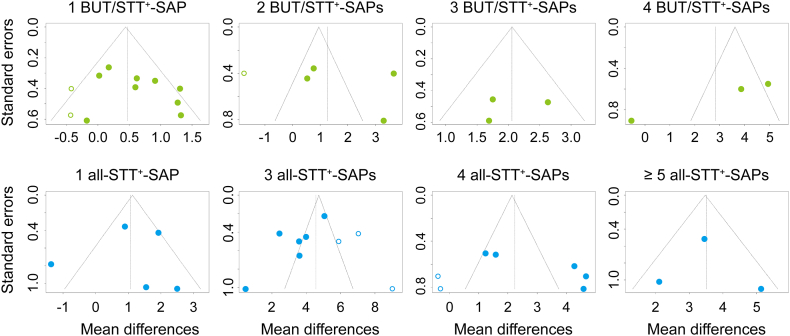
**Publication bias for BUT and STT in the subgroups based on the number of SAPs.** Publication bias for BUT (upper) and STT (lower) was assessed by visual inspection of funnel plots with Egger's test. Filled and open circles are for the RCTs analyzed and studies added by trim and fill analysis, respectively.

All RCTs were sub-grouped based on the number of SAPs, and the subgroup meta-analyses were examined for the outcomes of BUT or STT in the acupuncture experimental group versus the control (Fig. 4, Fig. 5). The subgroups for BUT included none to four of five BUT/STT^+^-SAPs in the experimental group, and the subgroups for STT included one to five (#1a and #17) and seven (#8) of nine SAPs positive on the STT (all-STT^+^-SAPs). Because the number of subgroups including five and seven all-STT^+^-SAPs, the outcomes of STT were analyzed in the subgroups including more than five all-STT^+^-SAPs. The outcomes of BUT were significantly increased in the subgroups including one to four BUT/STT^+^-SAPs, compared with the control (Fig. 4). The outcomes of STT were significantly increased in the subgroups including more than three all-STT^+^-SAPs compared with the control (Fig. 5). The sensitivity analyses showed changes of the significant outcomes for BUT only in the subgroups including two and four BUT/STT^+^-SAPs, but no changes of them in the other subgroups (Fig. 6A). There were no changes of the significant outcomes for STT in all of the subgroups (Fig. 6B). Publication bias showed asymmetry only in the outcomes of BUT in the subgroups including 4 BUT/STT^+^-SAPs (Fig. 7). Thus, the validated positive effects on BUT and STT were found in the subgroups including three BUT/STT^+^-SAPs and more than three all-STT^+^-SAPs, respectively, and the MDs of the subgroups were higher than those of all 21 RCTs.

### 3.6 SAP-depending improvements of BUT and STT in different acupuncture protocols

**Fig. 8:**
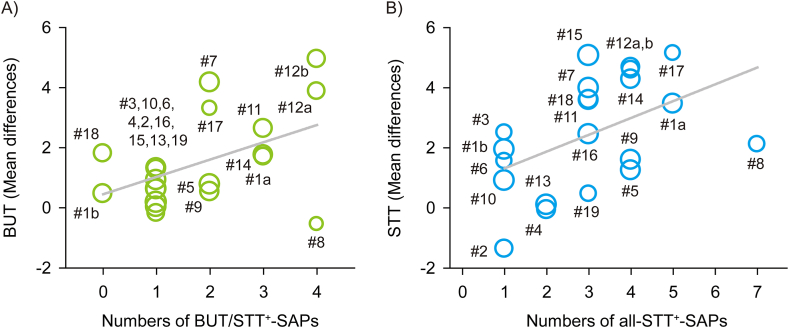
**Meta-regression analysis on BUT and STT in subgroups based on the number of SAPs.** Meta-regression alaysis was examined for correlations between the number of SAPs and outcomes of BUT (A) or STT (B). Circles with the numbers indicate the RCTs in the subgroups based on the number of SAPs, and the circle sizes correspond to the weights. A significance is indicated as a line.

Meta-regression analysis revealed significant correlations between the number of SAPs and the outcomes of BUT (Fig. 8A) and STT (Fig. 8B) in all acupuncture protocols of 21 RCTs (*P* < 0.01). Both BUT and STT were also improved more as the more SAPs were included in subgroups of the short-term acupuncture at all frequencies or low-frequencies (*P* < 0.05, Table 4). In addition, the SAP-depending improvements of BUT only were observed in subgroups of the low-frequency acupuncture for all treatment periods (*P* < 0.01). However, the SAP-depending improvements of both BUT and STT were not observed in subgroups of the high-frequency acupuncture for all treatment periods or short-term, and the long-term acupuncture at all frequencies or low-frequencies.

**Table 4:** Meta-regression analysis on BUT and STT in subgroups based on the number of SAPs in different acupuncture protocols.

Covariates	Tear-film breakup time (BUT)			Schirmer tear test (STT)		
	coefficient	R^2^ (%)	p-value	coefficient	R^2^ (%)	p-value
All treatment periods (21–90 days)						
all freq. (2.0–7.0 s/w)	0.65	40.02	0.00	0.58	19.91	0.03
high-freq. (2.0–3.0 s/w)	−0.11	0.00	0.82	0.46	20.48	0.14
low-freq. (3.7–7.0 s/w)	0.97	76.89	0.00	0.77	17.60	0.08
***Short-term (21–30 days)***						
all freq. (2.0–7.0 s/w)	0.89	46.65	0.00	0.86	34.16	0.01
high-freq. (2.0–3.0 s/w)	0.89	11.93	0.24	0.66	18.94	0.18
low-freq. (3.7–7.0 s/w)	1.06	71.35	0.00	1.12	28.80	0.04
***Long-term (51–90 days)***						
all freq. (2.0–7.0 s/w)	0.05	0.00	0.84	0.38	0.00	0.43
high-freq. (2.0–3.0 s/w)	–	–	–	–	–	–
low-freq. (3.7–7.0 s/w)	0.47	54.29	0.11	0.38	0.00	0.61

Meta-regression analysis was examined for correlations between the number of SAPs and outcomes of BUT and STT in subgroups of different acupuncture protocols based on the treatment days and frequencies. Frequencies (freq.) were expressed as sessions per week (s/w).

**Fig. 9:**
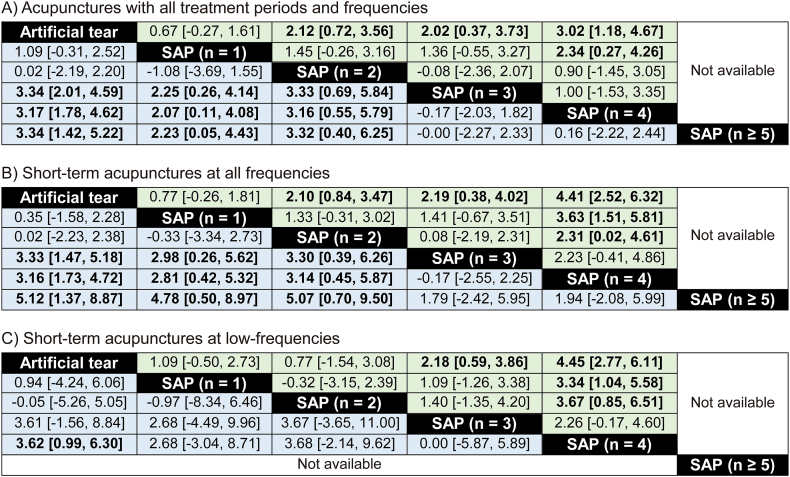
**Network meta-analysis for BUT and STT in SAP-depending acupuncture protocols.** Network meta-analysis was examined for correlations between the number of SAPs and outcomes of BUT (light green) and STT (light blue) in subgroups of the different acupuncture protocols in the RCTs. The acupuncture protocols were divided by all treatment periods and frequencies (A), short-term at all frequencies (B), and short-term at low-frequencies (C). The results are expressed as posterior medians with 95% credible intervals in brackets, and the column is compared with the row. Bold means significances. (For interpretation of the references to colour in this figure legend, the reader is referred to the Web version of this article.)

**Fig. 10:**
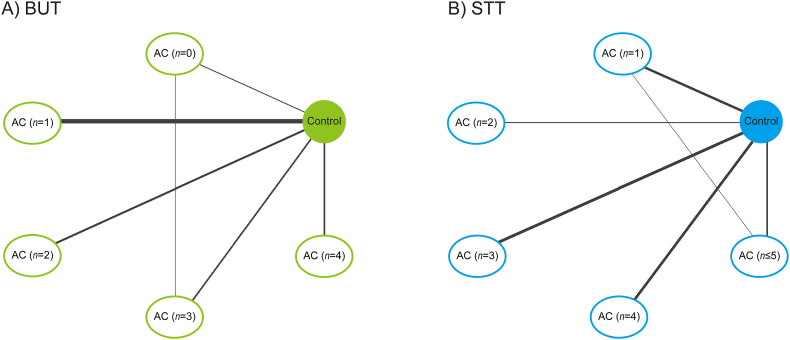
**Network diagram in network meta-analyses for BUT and STT in subgroups of SAP-depending acupuncture.** In network diagram for BUT (A) and STT (B), circles indicate the control and acupuncture (AC) subgroups based on the number of SAPs, and the line thickness corresponds to the number of RCTs.

**Fig. 11:**
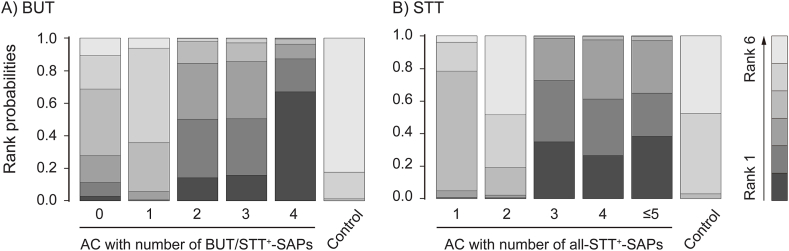
**Rankogram of network meta-analyses for BUT and STT in subgroups of SAP-depending acupuncture.** Rank probabilities for BUT (A) and STT (B) were examined in in acupuncture (AC) subgroups based on the number of SAPs.

Network meta-analyses showed significant improvements of both BUT and STT in the subgroups of all acupuncture protocols (Fig. 9A) or short-term acupuncture at all frequencies with more than three SAPs, compared with those in the artificial tear control (Fig. 9B). In network diagrams of the network meta-analyses for BUT (Fig. 10A) and STT (Fig. 10B), there were direct evidences between the control and the acupuncture subgroups based on the number of SAPs in all acupuncture protocols. The competing interventions were found only between the subgroups including none and three SAPs for BUT and between the subgroups including one and five SAPs for STT. The transitivity assumption showed no significant differences in the competing interventions for BUT (*P* = 0.71) and STT (*P* = 0.58) between the direct and indirect evidences, indicating the validity of network meta-analyses. The rank probabilities showed that the optimal numbers of SAPs can be four for BUT (Fig. 11A) and more than three for STT (Fig. 11B). In the network meta-analyses, the improvements of both BUT and STT were greater in the subgroups of all acupuncture protocols with four SAPs than in those with one (Fig. 9A), and also greater in the subgroups of short-term acupuncture at all frequencies with four SAPs than in those with one or two (Fig. 9B). In addition, the SAP-depending improvements of both BUT and STT were greater in the subgroups of short-term acupuncture at low-frequencies with four SAPs than the control (Fig. 9C). The values versus the control were similar or higher at low-frequencies than at all frequencies, in the subgroups of short-term acupuncture with four SAPs.

## 4 Discussion

Various combinations using 31 acupoints were applied in the acupuncture protocols among 21 RCTs analyzed in this study, and the complex combinations made it difficult to determine which acupoints were critical to treating DES. Furthermore, since there have been a few studies on the differential effects of acupoints in DES, an indirect comparison of Bucher analysis was used to examine acupoints improving the primary objective outcomes of BUT and STT. The subgroup analyses identified nine SAPs, and the positive relations of two SAPs with the positive effects on both BUT and STT suggested the potential combinations of ‘KI3–LI4–SP6’, ‘KI3–GB14–ST2’, or ‘KI3–BL1–EX-HN7–SP6’. The subgroup meta-analyses showed significant improvements of BUT or STT in the RCTs including more than three SAPs, and the meta-regression and Bayesian network meta-analyses validated the results with consistent stabilities. These analyses demonstrated that short-term (21–30 days) acupunctures including four SAPs, particularly at low-frequencies (two–three times/week, up to 13 sessions) can be optimal for treating typical DES. Although the indirect analysis requires more caution than a direct comparison, the Bucher analyses combined with network meta-analysis may allow TCM practitioners to improve the therapeutic effects [28].

Therapeutic improvements of DES are influenced by stimulating the relevant acupoints; however, the potential acupoints are different even between TCM and traditional Korean medicine (KM) based on TCM: TCM uses combinations of 11 main acupoints of ‘BL1/2, EX-HN5, GB14/20/37, LI4, LR3, SP6, ST2, and TE23’ with 12 adjunct points depending on the conditions [64,65], while KM employs only four same acupoints of ‘BL2, GB14/20, and TE23’ and seven different points, ‘EX-HN1, GV23, Heart (HT)8, LU9/10, SP3, and ST1’ [66]. All of 11 main acupoints in TCM and only one different main acupoint, ‘ST1’, in KM were available in these analyses, because 20 of 21 RCTs (except for #4) were conducted by Chinese practitioners. These subgroup analyses showed that the SAPs involved in improvements of BUT or STT were five main acupoints of ‘BL1, GB14, LI4, SP6, and ST2’ and four different acupoints of ‘EX-HN7, GV20, KI3, and ST36’ in TCM. The acupunctures using the other six main acupoints, excepting ‘GB37’, were involved in the negative effects on both BUT and STT. Although the different acupoints are not mainly applied for DES in TCM, ‘EX-HN7’ combined with ‘BL1 and GB14’ is commonly used to treat various eye diseases besides DES; ‘ST36’ as well as ‘LI4, SP6, and ST2’ are selected to promote *Qi* and blood circulation for relieving the eye pain; *Yin* meridians including KI can be activated by simulating at ‘SP6’ [7,67]. In agreement with these results, one of previous meta-analysis studies has reported that acupuncture on ‘ST2’ and ‘BL2 and ST1’ can be positive and negative, respectively, on improvements of BUT and STT in DES [12]. Another meta-analysis study has shown similar potentials of ‘LI4’ in DES, but different results that ‘ST1 and TE23’ can be the optimal acupoints [13]. The inconsistent results may be due to the different RCTs included in the meta-analyses. Further comprehensive studies are needed to clarify whether acupuncture on the SAPs effectively treats the symptoms of DES.

Tear secretion is regulated by complex processes of the lacrimal functional unit through an activation of (1) afferent nerves on the ocular surface, (2) innervating nerves in the lacrimal gland, and (3) lacrimal gland secretion [68]. The lacrimal gland is mainly activated by a trigeminal-parasympathetic reflex and mediated by the facial nerve [69]. However, a disturbance of the lacrimal functional unit can reduce the lacrimal secretion and damage the ocular surface, leading to a stimulation of the corneal nerve fibers with the peripheral and central nerves [68,70]. The periocular SAPs are correlated with the certain trigeminal nerve innervations [59,60]: ‘BL1’ and ‘GB14’ are correlated with the infratrochlear nerve (of the nasociliary nerve) and the supraorbital nerve branch (of the frontal nerve), respectively, in V1; ‘ST2’ and ‘EX-HN7’ are correlated with the infraorbital nerve and its branch (the lateral branch of the inferior palpebral nerve), respectively, in V2. The head point of ‘GV20’ is located at the frontal sensory nerve branches in V1. The other periocular acupoints with the negative effects on both BUT and STT are correlated with the different trigeminal nerve innervations, although some of them target the similar nerve branches. In this context, acupuncture on the periocular SAPs may improve the symptom of DES by stimulating their own trigeminal V1 and V2 branches. Furthermore, a stimulation of the trigeminal sensory nerve via an interaction with the facial nerve increases the cerebral blood flow [71]. The periocular SAPs of ‘EX-HN7, GB14, and ST2’ and the distant SAP of ‘LI4’ are indeed selected to treat Bell's palsy with the facial nerve injuries for enhancing the facial blood flow and improving function of the facial nerve [63,72]. A stimulation on the local SAP of ‘GV20’ is also known to enhance the cerebral blood flow and neuronal activities in the central and peripheral nerves [73]. The exact mechanisms are unclear; however, typical DES may be improved by stimulating the SAP-dependent neuronal regulation and vasodilation.

The periocular acupoints were frequently applied in the acupuncture of 21 RCTs; however, four of nine SAPs were distant points far from the symptomatic orbit. Furthermore, among 11 combinations of two SAPs with the positive effects on both BUT and STT, four and five combinations were two distant SAPs and each of the local and distant SAPs, respectively. On the other hand, three combinations with the negative effects on both BUT and STT had either of two local or two distant SAPs. It suggests benefits of the distant acupoints and an importance of harmony with the local and distant acupoints. Traditional acupuncture indeed uses acupoint combinations of local symptomatic points with distant points from the painful sites [64,65]. Wei et al. [14] consistently reported that acupuncture on both periocular and distant acupoints is more effective than that on periocular acupoints alone in treating DES. Herein, the positive relations in improvements of both BUT and STT were found in acupoint combinations with all distant SAPs (‘KI3–LI4–SP6’) or local and distant SAPs (‘KI3–GB14–ST2’ and ‘KI3–BL1–EX-HN7–SP6’). The distant SAPs of ‘KI3, LI4, and SP6’ are commonly used in managements of pain syndrome [18,74] or peripheral neuropathy [63]. It is believed that pain modulation through an acupuncture on distant acupoints can assist the peripheral mechanisms of local acupoints by vasodilation and segmental inhibition [75,76]. A few studies have reported the differential effects of acupoints: acupuncture on the periocular SAP of ‘BL1’ improves tear secretion in DES and the visual function [77], while stimulating distant SAPs of ‘LI4’ and ‘SP6 and ST36’, produce analgesic effects by coordinating with the central nervous system and by releasing opioid peptides and inhibiting the inflammation, respectively [18,78,79]. The distant SAPs are known to have analgesic and anti-inflammatory effects by activating the vagus nerve [80] or alleviating the central sensitization [81,82]. Acupuncture on KI3 is reported to stimulate the visual-associated cortex and improve the visual function in cognitive disorders [83,84]. Although it is unclear how distant acupoints regulate symptoms of DES, at least the releases of opioid peptides may account for the analgesic effects and contribute to balance the neurotransmitters or blood flows.

These meta-analyses demonstrated the SAP-depending improvements of BUT and STT in a short-term (≤30 days) acupuncture with up to 13 sessions, and effects of the acupuncture protocol on BUT and STT were reviewed in the other clinical studies not included in these meta-analyses. Tong et al. [44] conducted the RCT with eight acupuncture sessions for 28 days on eight acupoints of five periocular non-SAPs (‘ST1, BL2, GB20, EX-HN5, and three tear needles’) and three distant SAPs (‘LI4, SP6, and ST36’). The acupuncture was superior to the artificial tear control in inhibiting the inflammation and improving the symptom scores, but not in improving the BUT and STT, suggesting beneficial effects of the periocular and distant SAPs on lacrimal secretion and anti-inflammation/antipain, respectively. Gong et al. [85] conducted a parallel comparative study with 10 acupuncture sessions for 21 days on 10 points including five SAPs of periocular ‘BL1, GB14, and ST2’ and distant ‘LI4 and SP6’. The outcomes of BUT were significantly lower in the acupuncture (0.4 ± 1.0 s) than the artificial tear control (1.6 ± 1.8 s) at 1 h after the treatments, and the outcomes of STT were similar between the acupuncture (1.6 ± 2.2 mm) and control groups (2.0 ± 3.0 mm). However, in the follow-up at 3 weeks after the treatments, the outcomes of BUT (0.4 ± 1.0 s) and STT (1.8 ± 2.2 mm) were non-significantly and significantly increased, respectively, in the acupuncture compared with the control (0.1 ± 0.4 s for BUT and 0.1 ± 1.3 mm for STT). Given that the applied five SAPs showed many positive relations on improvements of STT in Fig. 3, the acupuncture may have the cumulative effects on the symptoms of DES.

Follow-up data in 21 RCTs were excluded here; however, two follow-up evaluations (#4 and #5) were compared between the acupuncture experimental group and the artificial tear control. One RCT (#4) conducted acupuncture with 12 sessions for 28 days on 17 points including bilateral two SAPs of periocular ‘GB14’ and distant ‘LI4’. The acupuncture significantly improved the BUT, OSDI, and VAS in the right eye, but not improved the BUT in the left eye and STT in both eyes, in the follow-up at 8 weeks after the treatments, compared with the control [43]. Another RCT (#5) conducted acupuncture with nine sessions for 21 days on seven points including four SAPs of periocular ‘BL1’, head ‘GV20’, and distant ‘LI4 and ST36’ [86]. The acupuncture significantly improved the STT (5.4 ± 2.1 mm) but not the BUT (1.6 ± 0.2 s) in the follow-up at one week, compared with the control (1.5 ± 0.3 s for BUT and 4.2 ± 1.8 mm for STT). Interestingly, four SAPs used had the negative and positive relations involved in improvements of BUT and STT, respectively, in Fig. 3, although the effects of acupuncture on BUT were similar with those of the control. It suggests that acupuncture protocol of the short-term at low-frequencies may require more SAPs with the positive relations on both BUT and STT in the harmonized combination of the local and distant points or *Yang* and *Ying* meridians for the long-term effectiveness in DES. Future follow-up study is necessary to evaluate the cumulative effects of acupuncture for the ideal therapeutic protocol.

This study has several limitations as follows: the analyzed RCTs have no double-blinded treatments and sham acupuncture to mitigate the placebo effects, and patient characteristics of their age, sex, and severity of DES were not considered. The main reason was because of small sample sizes for analyzing them. Furthermore, it is difficult to achieve double-blindness in acupuncture studies requiring one TCM practitioner for similar stimulation and sensation on secured acupoints. The placebo effects using sham acupuncture is still controversial because sham acupuncture may not provide sufficient sensitivity, or needle-insertion on non-acupoints close to active points may induce unexpected physiological responses compared with that on less painful acupoints [29,87]. There have been two placebo-controlled trials comparing the effectiveness of acupuncture with the placebo control using non-acupoints in patients with typical DES [66] or all types of DES including Sjögren's syndrome and rosacea [88]. Compared with the sham control, the BUT and STT were not improved in the acupuncture with nine sessions for 21 days on 11 points including one periocular SAP of ‘GB14’ up to the follow-up at four weeks [66], or in the acupuncture with two sessions for two days on six points not analyzed here in the follow-up at six months [88]. It is assumed that acupunctures on the acupoint combinations may have similar effects on the BUT and STT with those of the placebo group or insufficient effects to improve the symptoms. This is the first meta-analysis study reporting the optimal acupuncture protocol to treat typical 10.13039/501100015193DES, supported by stable and consistent data in the subgroup and network meta-analyses. However, there was high heterogeneity across the RCTs probably due to the small sample sizes or the practitioner-dependent needling in the orbital region with complicated and high-density nerve and vascular elements. Thus, prospective clinical trials with large sample sizes should be accomplished to clarify the optimal acupuncture protocol for typical DES. These results provide useful information for guiding acupuncture in clinical trials for DES.

## Authors contribution statement

Joon-Gon Park: Data curation, formal analysis, investigation, methodology, software, resources, validation, visualization, and writing - original draft. Bong Hyo Lee: Methodology, validation and writing-review & editing. Ji-Ho Na: Formal analysis and resources. Ji-Hyeo Jung: Formal analysis and resources. Chang-Hyun Song: Conceptualization, data curation, formal analysis, funding acquisition, investigation, methodology, supervision, project administration, resources, validation, visualization, writing - original draft, and writing-review & editing.

## Funding statement

This work was supported by the 10.13039/501100003725National Research Foundation of Korea (NRF), South Korea grant funded by the Korea government (MSIP) (No. 2018R1A5A2025272) and the Basic Science Research Program through the NRF funded by the Ministry of Education (No. 2020R1I1A3A04037939).

## Data availability statement

All data generated or analyzed during this study are included in the article and its supplementary information files.

## Declaration of interest's statement

The authors declare no conflict of interest.

## Declaration of competing interest

The authors declare that they have no known competing financial interests or personal relationships that could have appeared to influence the work reported in this paper.
